# [Retracted] Establishment of ApoE-knockout mouse model of preeclampsia and relevant mechanisms

**DOI:** 10.3892/etm.2025.13000

**Published:** 2025-10-23

**Authors:** Wenjuan Sun, Baoxia Cui, Fanzhen Hong, Yongping Xu

Exp Ther Med 12:2634–2638, 2016; DOI: 10.3892/etm.2016.3678

Following the publication of this paper, it was drawn to the Editor’s attention by a concerned reader that certain of the images showing structural changes in the placenta in Fig. 3 on p. 2637 were strikingly similar to data that had already been published in different form in another article written by different authors at a different research institute in the journal *PLoS One*. Moreover, within Fig. 3B itself, certain of the data were strikingly similar to data that appeared in another region of the same panel, suggesting that the presentation of these data may have been anomalous.

Owing to the fact that the contentious data in the above article had already been published prior to its submission to *Experimental and Therapeutic Medicine,* the Editor has decided that this paper should be retracted from the Journal. The authors were asked for an explanation to account for these concerns, but the Editorial Office did not receive a reply. The Editor apologizes to the readership for any inconvenience caused.

